# Host Genetic Factors, Comorbidities and the Risk of Severe COVID-19

**DOI:** 10.1007/s44197-023-00106-3

**Published:** 2023-05-09

**Authors:** Dongliang Zhu, Renjia Zhao, Huangbo Yuan, Yijing Xie, Yanfeng Jiang, Kelin Xu, Tiejun Zhang, Xingdong Chen, Chen Suo

**Affiliations:** 1 Department of Epidemiology & Ministry of Education Key Laboratory of Public Health Safety, School of Public Health, Fudan University, Shanghai, China; 2 Shanghai Institute of Infectious Disease and Biosecurity, Shanghai, China; 3 State Key Laboratory of Genetic Engineering, Zhangjiang Fudan International Innovation Center, Human Phenome Institute, Fudan University, Shanghai, China; 4 Fudan University Taizhou Institute of Health Sciences, Yaocheng Road 799, Taizhou, Jiangsu China; 5 Department of Biostatistics, School of Public Health, Fudan University, Shanghai, China; 6 National Clinical Research Center for Aging and Medicine, Huashan Hospital, Fudan University, Shanghai, 200040 China; 7 Yiwu Research Institute of Fudan University, Yiwu, Zhejiang China

**Keywords:** COVID-19, GWAS, Host genetics, Comorbidity, Polygenic risk score, Predictive model

## Abstract

**Background:**

Coronavirus disease 2019 (COVID-19), caused by severe acute respiratory syndrome coronavirus 2 (SARS-CoV-2), was varied in disease symptoms. We aim to explore the effect of host genetic factors and comorbidities on severe COVID-19 risk.

**Methods:**

A total of 20,320 COVID-19 patients in the UK Biobank cohort were included. Genome-wide association analysis (GWAS) was used to identify host genetic factors in the progression of COVID-19 and a polygenic risk score (PRS) consisted of 86 SNPs was constructed to summarize genetic susceptibility. Colocalization analysis and Logistic regression model were used to assess the association of host genetic factors and comorbidities with COVID-19 severity. All cases were randomly split into training and validation set (1:1). Four algorithms were used to develop predictive models and predict COVID-19 severity. Demographic characteristics, comorbidities and PRS were included in the model to predict the risk of severe COVID-19. The area under the receiver operating characteristic curve (AUROC) was applied to assess the models’ performance.

**Results:**

We detected an association with rs73064425 at locus 3p21.31 reached the genome-wide level in GWAS (odds ratio: 1.55, 95% confidence interval: 1.36–1.78). Colocalization analysis found that two genes (SLC6A20 and LZTFL1) may affect the progression of COVID-19. In the predictive model, logistic regression models were selected due to simplicity and high performance. Predictive model consisting of demographic characteristics, comorbidities and genetic factors could precisely predict the patient’s progression (AUROC = 82.1%, 95% CI 80.6–83.7%). Nearly 20% of severe COVID-19 events could be attributed to genetic risk.

**Conclusion:**

In this study, we identified two 3p21.31 genes as genetic susceptibility loci in patients with severe COVID-19. The predictive model includes demographic characteristics, comorbidities and genetic factors is useful to identify individuals who are predisposed to develop subsequent critical conditions among COVID-19 patients.

**Supplementary Information:**

The online version contains supplementary material available at 10.1007/s44197-023-00106-3.

## Introduction

Coronavirus disease 2019 (COVID-19), caused by the novel severe acute respiratory syndrome coronavirus 2 (SARS-CoV-2), has infected over 630 million people and resulted in 6 million deaths as of 11 November 2022 [1]. Epidemiological data and clinical records have shown high heterogeneity of COVID-19, with a wide spectrum of clinical symptoms varying from asymptomatic, mild to moderate, severe, and critical conditions [2, 3]. Although only a small proportion of cases with critical conditions (5%) [4], they will contribute to a considerable number of individuals at high risk of death due to the large number of infections in total. Mortality is primarily attributed to patients with severe and critical conditions, such as severe respiratory failure associated with interstitial pneumonia and acute respiratory distress syndrome [5]. Patients with severe COVID-19-related respiratory failure usually require prolonged mechanical ventilatory support [6].

Although the pathogenesis of severe COVID-19 and related respiratory failure is unclear, previous studies have reported many key factors associated with COVID-19 severity, including demographic characteristics such as age, gender, BMI, and socioeconomic status, and comorbidities such as chronic kidney disease, chronic lung disease, cardiovascular disease, diabetes, and cancer [7–11]. Additionally, a series of genome-wide association studies (GWASs) have demonstrated the crucial role of host genetic factors in modulating the risk of infection and disease severity [8, 12–14], especially single nucleotide polymorphisms (SNPs) on immune-related genes, such as TLR7, IFNAR2, and IL10RB [15, 16]. These SNPs provided quantitative measures of genetic susceptibilities and contribute to population stratification. Based on the results of GWAS, polygenic risk scores (PRSs) could be calculated and applied to help identify individuals at high risk of specific diseases as the highly polygenic of genetic architecture [17].

However, there are limited studies considering the joint effect of genetic and non-genetic factors in predicting the severity of COVID-19. And most severe COVID-19-related GWASs used uninfected populations as control [14, 16, 18, 19], which could not reflect the difference between severe and non-severe patients among those with infection. Therefore, in this study we sought to evaluate host genetic factors focusing on severe COVID-19, to explore the association of comorbidities and COVID-19 severity, and to predict individual predisposition for adverse prognosis after infection.

## Methods

### Study Population

**Fig. 1:**
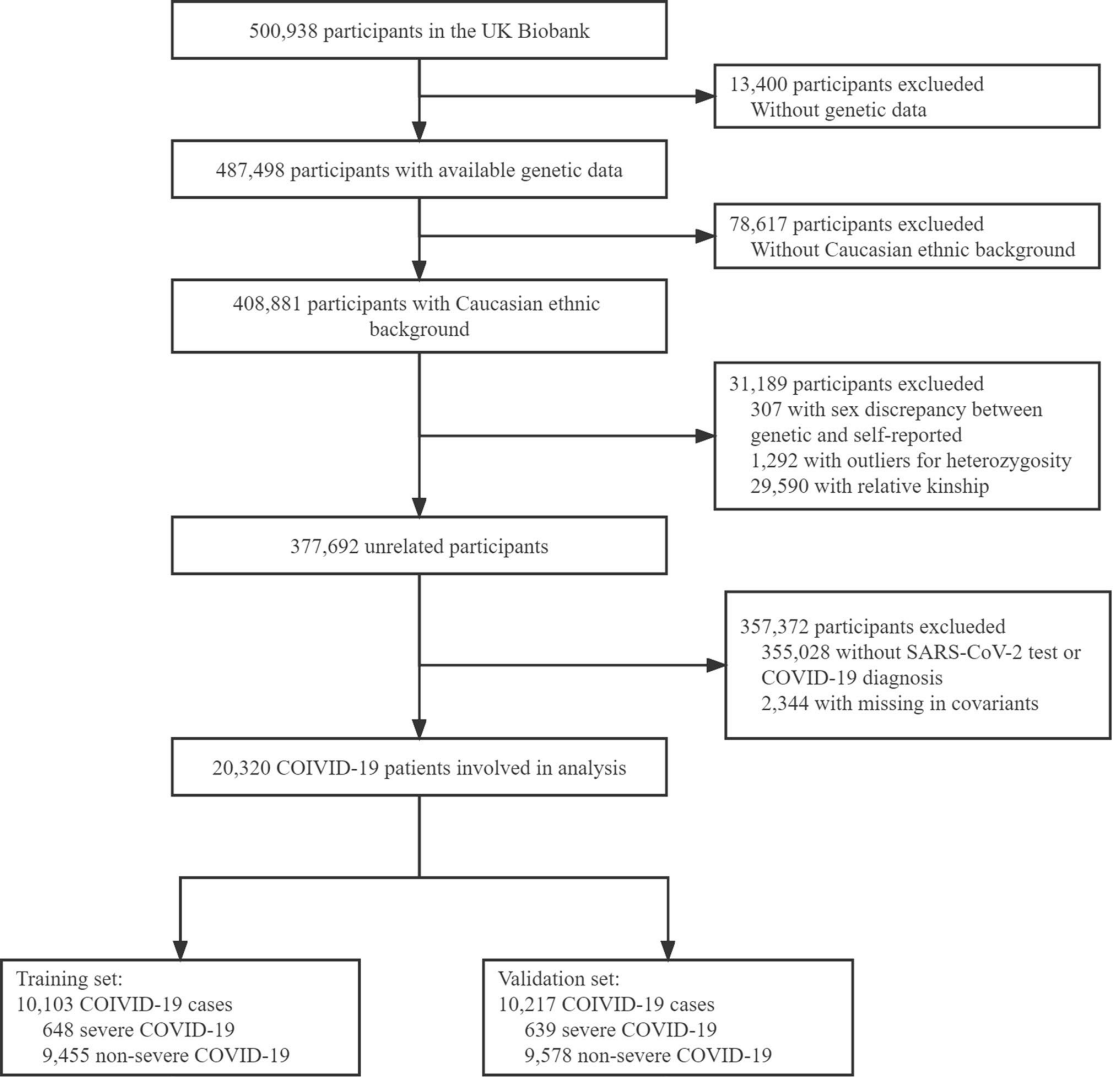
The flowchart for the selection of study participants from the UK Biobank cohort

We include individuals with COVID-19 based on UK Biobank (UKB) study, a large prospective cohort study involving over 0.5 million participants aged 40 to 69 between 2006 and 2010 with comprehensive phenotyping and genomic data. Details of the design and method of UKB have been described previously [20]. The flowchart of the selection of study samples was shown in Fig. 1. Briefly, we involve all COVID-19 cases that passed the GWAS quality control procedure with Caucasian ethnic backgrounds.

### Definition of Outcome

COVID-19 cases were identified according to qPCR testing or the International Classification of Diseases, Tenth Revision (ICD-10) for COVID-19-related diagnoses. We defined individuals who met any of the following criteria as COVID-19 cases [14]: (1) a positive qPCR for SARS-CoV-2; (2) COVID-19-related inpatient diagnosis (ICD-10: U071, U072 and U073 in variable ‘diag_icd10’ in table ‘hesin_diag’); (3) COVID-19 related death (ICD-10: U071, U072 and U073 in variable ‘cause_icd10’ in table ‘death_cause’). Based on the above criteria, severe cases need to fulfill additional criteria: (1) respiratory support in the hospital (ICD-10: Z998); (2) respiratory support during operation (ICD-10: E85, E87, E89, X56, and X58); (3) COVID-19 related death (ICD-10: U071, U072 and U073).

### Demographic Characteristics and Comorbidities

We evaluated participants’ demographic characteristics and comorbidities. Considering the influence of economic status on health care and the severity of COVID-19, average annual household income assessed at recruitment was involved as a covariate and categorized into five groups: < £18,000, £18,000–30,999, £31,000–51,999, £52,000–100,000 and > £100,000. We introduced the Charlson Comorbidity Index (CCI) to account for individuals’ comorbidities before COVID-19 onset [21, 22]. Chronic diseases in cerebrovascular, cardiac, pulmonary, hepatic and renal, as well as diseases affecting systemic function such as diabetes, paraplegia, dementia, malignancy and AIDS were included in CCI. Details about components of CCI were shown in supplement Table S1. The conditions in CCI were confirmed ICD-10 in the UK Biobank inpatient hospital data. All conditions were diagnosed before Jan 1, 2020, when COVID-19 began. The distribution of CCI for each participant was shown in Fig S1. We defined individuals with no more than one mild comorbidity (CCI score ≤ 1) as the low CCI group, and participants with a CCI score > 1 as the high CCI group, which indicates the individual had at least one severe comorbidity. Other characteristics such as age, gender and BMI were assessed at recruitment as described in previous studies [20]. Individuals with missing values in age or gender were excluded from our analysis. Missing values in other covariates (proportions range from 2.5 to 11%) were imputed with median value.

### Genetic Association Analyses for Severe COVID-19

Genotyping data in the UK Biobank were derived from the GWAS chip (Affymetrix UK BiLEVE and UK Biobank Axiom arrays) using blood samples collected at baseline for each participant. These genotyping data were imputed using reference panels of the Haplotype Reference Consortium, or UK10K, and 1000 Genomes Project phase 3 [23]. We then applied filters to achieve high-quality variants with (1) INFO score (information metric)  ≥ 0.5; (2) call rate  ≥ 99%; (3) minor allele frequency (MAF)  ≥ 1%; (4) Hardy–Weinberg equilibrium (HWE) ≥ 1 × 10^–6^. We further excluded variants in the MHC region (chr6 25-35 Mb) due to extensive linkage disequilibrium (LD). The final set of data contained a total number of 8,378,356 variants. Firth logistic regression test was implemented in PLINK (version 1.9) to test the association of single nucleotide polymorphisms (SNPs) and phenotype [24, 25]. Age, gender, genotyping array and first 10 principal components (PCs) were adjusted for population heterogeneity in the multi-variable regressions. We additionally performed a GWAS in participants low CCI group, each severe COVID-19 patient was matched with four non-severe COVID-19 control by gender and age, that’s 3,860 participants in total (772 cases and 3,088 controls).

Independent significant SNPs were extracted when their P-values reach genome-wide significant threshold (*P* ≤ 5.0 × 10^–8^) and in low LD (*r*^2^ < 0.4) with other SNPs within a 500-Kb window. Lead SNPs were identified as a subset of the independent significant SNPs with the lowest P-values and were in LD with each other at *r*^2^ < 0.1 within a 1-Mb window.

### Colocalization of cis-eQTL and COVID-19 GWAS Signals

Since cis-regulation of gene expression is a common pathway for genetic variation to affect complex diseases [26], expression trait loci (eQTL) mapping could be used to identify candidate genes for traits or diseases of interest [27]. To explore the association between COVID-19 GWAS signals and gene expression, we performed a colocalization analysis of cis-eQTL and COVID-19 GWAS signals. eQTL data were obtained from the GTEx Portal [28], including all SNP-gene association tests, either significant or non-significant in all GTEx V8 tissues of 838 post-mortem donors and gene-level information. For the COVID-19 GWAS significant signals (*P* ≤ 5.0 × 10^–8^), we expanded each variant’s position to a special locus by 500 kb upstream and downstream and located functional genes in this region.

We used the colocalization method in ‘Coloc’ (version 5.1.0) to evaluate the probability that the same signal can both modify the risk of severe COVID-19 and affect the expression level of a specific gene [29, 30]. ‘Coloc’ uses estimated approximate Bayes factors from summary association data to compute posterior probabilities (PPH4) assuming one causal variant per trait [31]. Colocalization was performed between genes’ cis-eQTL signals in each of 49 GTEx tissues and COVID-19 severity GWAS to find the candidate causal variants [29]. In the present study, PPH4 over 0.75 were considered as strong evidence for colocalization.

### Polygenetic Risk Score

For the calculation of PRS, we selected COVID-19 severity-related meta-GWAS summary published by HGI (A2 leave 23andme and UKBB, Release V7) to avoid possible overfitting [32]. Only biallelic SNPs with MAF > 5% were included in the PRS analysis. We derived independent SNPs (*r*^2^ < 0.1 within a 1-Mb window) associated with COVID-19 severity based on GWAS summary statistics at different P value threshold (5 × 10^–15^, 5 × 10^–10^, 5 × 10^–7^, 1 × 10^–5^, 0.001, 0.05). For each participant, PRS was calculated as the sum of risk alleles present at each locus, weighted by the odds ratio. We used Nagelkerke’s R^2^ and the number of SNPs for constructing PRS to select the most appropriate threshold via PRSice-2 (version 2.3.5) [33, 34]. The best-fitted PRS were applied as an indicator of genetic risks for severe COVID-19 in the following analysis. PRS was classified into low (bottom 50%) and high (top 50%) groups according to the quantile of PRS scores.

### Statistical Analysis

Characteristics of participants were described as means (standard deviations) or frequencies (percentages) in severe COVID-19 and non-severe COVID-19 patients. Associations of covariates and PRS with COVID-19 severity were analyzed in the logistic model. Genome-association analysis was performed in Plink adjusted with age, gender, genotyping array and first 10 PCs. Considering the potential confounding of commodities and age, we further assessed the association of lead SNPs with comorbidities and age in a multivariate logistic model.

All COVID-19 patients were randomly split into a train set and validation set (1:1). Setting severe COVID-19 as an outcome, we constructed predictive models using four different machine learning methods [Logistic regression, random forest, partial least squares (PLS) regression, and bagged flexible discriminant analysis (FDA)] [35–37]. Age, gender, income, BMI, CCI and PRS were involved as predictors in constructing the model. Ten-fold cross validation and the areas under the receiver operating characteristic curves (AUROCs) were used to measure the models’ performance in training set. The best-fit models in the training set were applied and validated in the validation set. AUROC, sensitivity and specificity were used to assess the performance of the model. The basic prediction model was constructed using age, gender, income as predictors, and other risk factors (BMI, CCI, and PRS) were added to the model, respectively. And a full model including all predictors above was constructed. All analyses were performed with R (version 4.2.0) software and ‘caret’ package.

## Results

### Study Participants

A total of 20,320 individuals who were confirmed with COVID-19 between Feb 21, 2020 and Mar 18, 2021, aged 50–83 years, were included in our analysis (Table 1). Of these, 1287 (6.33%) participants were identified as severe COVID-19 if COVID-19-related respiratory support or death occurred. The demographic characteristics and comorbidities were significantly different between severe and non-severe COVID-19 patients. Specifically, severe cases were more likely to be man, elder, with high BMI, low income, and high CCI. Age group over 80 had the highest odds ratio (OR = 21.8, 95%CI 16.8–28.2) in severe COVID-19 compared to non-severe cases, suggesting that age might play an important role in disease progression. We further compared the distribution of each item in constructing CCI and found all but AIDS diagnosed prior to COVID-19 were associated with severity (Table 2). Among the comorbidities, chronic pulmonary disease was the most common (22.1%) comorbidity among severe patients, followed by diabetes (19.7%). The details of the effect for each type of comorbidity could be found in Table 2. In short, CCI scores were a good representation of the underlying health status of the population, and individuals with more comorbidities had a higher risk of severe COVID-19. We categorized participants into low-CCI (CCI ≤ 1) and high-CCI (CCI > 1) groups, mainly considering the distribution and clinical practice (Fig S1).

**Table 1:** Demographic characteristics and comorbidities of patients with COVID-19

Characteristics	Non-severe COVID-19	Severe COVID-19	Odds Ratio (95%CI)^*^	*P* Value^*^
	(*N* = 19,033)	(*N* = 1,287)		
Gender (*n*, %)				
Female	10,212 (43.7)	450 (35.0)	*Reference*	
Male	8,821 (46.3)	837 (65.0)	2.15 (1.91,2.42)	< 0.001
Age (mean ± SD)	65.13 ± 8.26	73.24 ± 6.97	1.14 (1.13,1.15)	< 0.001
Age group (*n*, %)				
50 ~ 60	5,997 (31.5)	87 (6.8)	*Reference*	
60 ~ 70	6,618 (34.8)	224 (17.4)	2.33 (1.82,3.00)	< 0.001
70 ~ 80	5,715 (30.0)	754 (58.6)	9.09 (7.26,11.4)	< 0.001
Over 80	703 (3.7)	222 (17.2)	21.8 (16.8,28.2)	< 0.001
BMI (mean ± SD)	27.81 ± 4.77	30.12 ± 5.83	1.08 (1.07,1.10)	< 0.001
Income				
< 18,000	2,986 (15.7)	437 (34.3)	*Reference*	
~ 30,999	4,001 (21.0)	291 (22.6)	0.50 (0.42,0.58)	< 0.001
~ 51,999	7,222 (37.9)	426 (33.1)	0.40 (0.35,0.46)	< 0.001
~ 100,000	3,887 (20.4)	110 (8.5)	0.19 (0.16,0.24)	< 0.001
> 100,000	937 (4.7)	23 (1.8)	0.17 (0.11,0.25)	< 0.001
CCI group (*n*, %)				
Low	16,723 (87.9)	772 (60.0)	*Reference*	
High	2,310 (12.1)	515 (40.0)	4.83 (4.28,5.44)	< 0.001

*SD* standard deviation, *CI* confidence interval, *IQR* interquartile range, *BMI* Body mass index, *CCI* Charlson Comorbidity Index

*Logistic regression model was used to estimate the effect and P value of each factor

**Table 2:** The association of CCI components and severe COVID-19 in a multivariate logistic regression model

Diseases (Abbr. %)	All COVID-19 cases (*N* = 20,320)	Non-severe COVID-19 (*N* = 19,033)	Severe COVID-19 (*N* = 1,287)	OR (95% CI)*	*P*-Value^*^
Myocardial infarction (MI, %)	663 (3.3%)	523 (2.7%)	140 (10.9%)	4.32 (3.55, 5.25)	< 0.001
Congestive heart-failure (CHF, %)	309 (1.5%)	208 (1.1%)	101 (7.8%)	7.71 (6.03, 9.85)	< 0.001
Peripheral vascular disease (PVD, %)	418 (2.1%)	303 (1.6%)	115 (8.9%)	6.07 (4.85, 7.58)	< 0.001
Cerebrovascular disease (CVD, %)	585 (2.9%)	434 (2.3%)	151 (11.7%)	5.70 (4.69, 6.92)	< 0.001
Dementia (%)	120 (0.6%)	79 (0.4%)	41 (3.2%)	7.85 (5.39, 11.56)	< 0.001
Chronic pulmonary disease (CPD, %)	2,040 (10.0%)	1,755 (9.2%)	285 (22.1%)	2.80 (2.43, 3.22)	< 0.001
Rheumatic diseases (Rh, %)	356 (1.8%)	289 (1.5%)	67 (5.2%)	3.56 (2.71, 4.68)	< 0.001
Peptic ulcer disease (PUD, %)	423 (2.1%)	361 (1.9%)	62 (4.8%)	2.62 (1.99, 3.45)	< 0.001
Mild liver disease (MLD, %)	326 (1.6%)	257 (1.4%)	69 (5.4%)	4.14 (3.15, 5.43)	< 0.001
Diabetes (DM, %)	1,149 (5.7%)	895 (4.7%)	254 (19.7%)	4.98 (4.28, 5.81)	< 0.001
Hemiplegia/Paraplegia	144 (0.7%)	103 (0.5%)	41 (3.2%)	6.05 (4.19, 8.72)	< 0.001
Moderate or severe renal disease (SRD, %)	356 (1.8%)	242 (1.3%)	114 (8.9%)	7.55 (5.99, 9.50)	< 0.001
Diabetes with end-organ damage (DMD, %)	115 (0.6%)	73 (0.4%)	42 (3.3%)	8.76 (5.97, 12.86)	< 0.001
Any malignant tumor (MT, %)	1,387 (6.8%)	1,214 (6.4%)	173 (13.4%)	2.28 (1.92, 2.70)	< 0.001
Moderate or severe liver disease (SLD, %)	46 (0.2%)	29 (0.2%)	17 (1.3%)	8.77 (4.81, 16.01)	< 0.001
Metastatic solid tumor (ST, %)	206 (1.0%)	175 (0.9%)	31 (2.4%)	2.66 (1.81, 3.91)	< 0.001
AIDS/HIV	11 (0.1%)	9 (0.0%)	2 (0.2%)	3.29 (0.71, 15.24)	0.128

*Abbr* Abbreviation, *OR* odds ratio, *CI* confidence interval

*P value and OR were estimated in the logistic regression model, adjusted for age, sex, income, and BMI

### Genome-Wide Association Analysis of Severe COVID-19

**Fig. 2:**
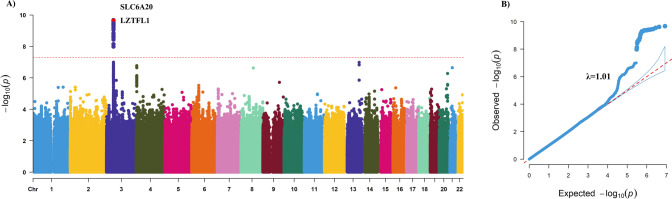
Result of Genome-wide association study on COVID-19 severity in UK Biobank cohort. **A**: Manhattan plot of severe COVID-19 GWAS highlighting susceptibility locus; **B**: Q-Q plot for severe COVID-19 GWAS

**Fig. 3:**
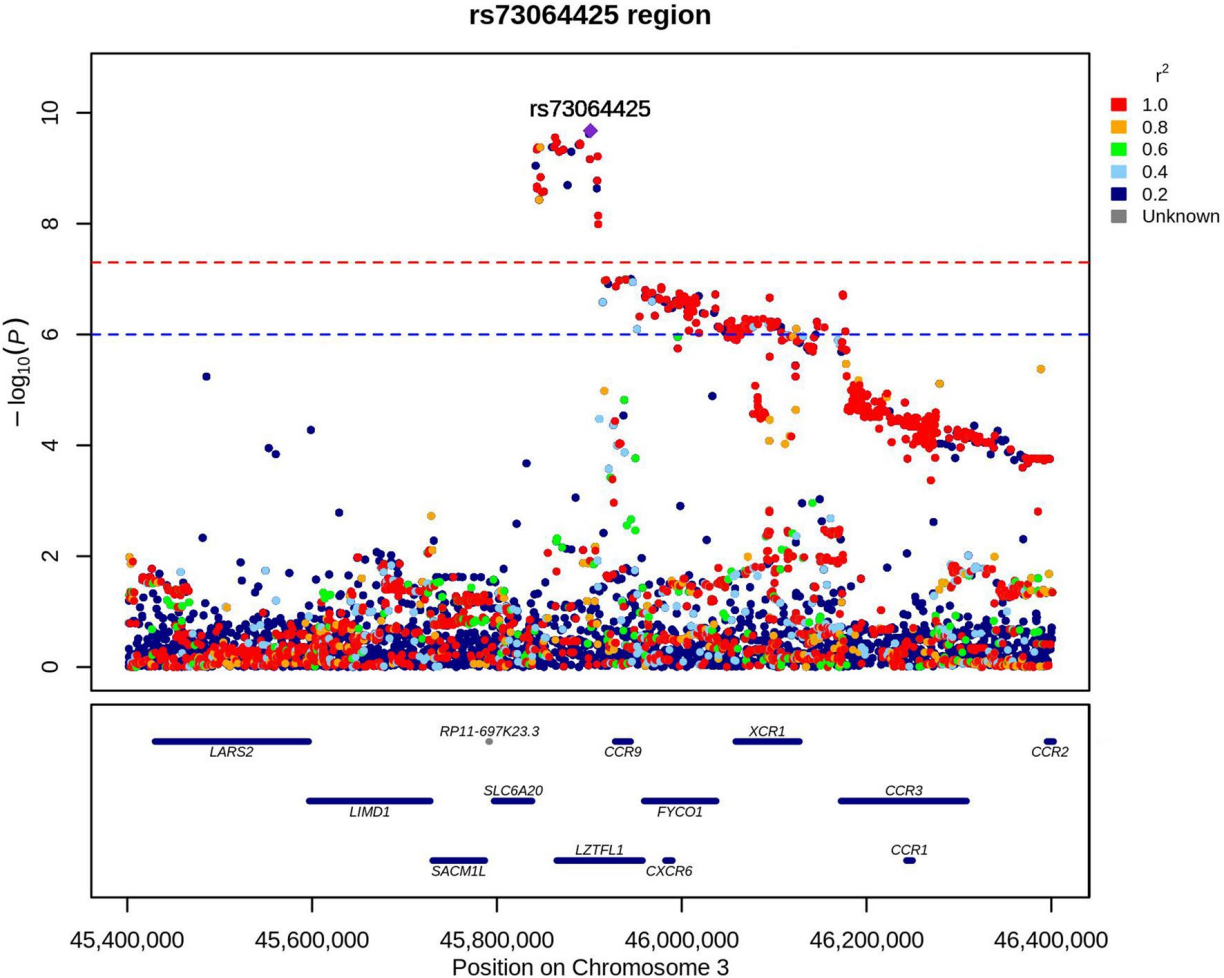
Locus zoom plot of rs733064425 locus (3p21.31) with 500-kb flanking region surrounding the lead SNP (rs733064425)

We performed GWAS of severe COVID-19 using 1,287 participants with severe COVID-19 and 19,033 participants with non-severe COVID-19 in the dataset of the UK Biobank study. Only one lead SNP, rs73064425 was found to be associated with COVID-19 severity at a significance level of *P* < 5 × 10^–8^ (Fig. 2A). Genomic inflation factor (λ_GC_) was estimated as 1.01, suggesting the well control of GWAS quality and no substantial impact of systematic inflation (Fig. 2B) [38]. We additionally added CCI into a logistic model for the lead SNPs to adjust the potential confounding of critical illness and the results remained robust. The most significant signal was rs73064425 T/C (OR = 1.55, 95%CI 1.36–1.78) at locus 3p21.31. Then, independent significant SNP was annotated with resided or nearby functional genes in upstream and downstream ± 500 Kb regions. The most significant SNP rs73064425 was found located at a region comprising six genes, SLC6A20, LZTFL1, CCR9, FYCO1, CXCR6, and XCR1 (Fig. 3). Further, the association between lead SNP (rs73064425) with age and comorbidities were assessed in the multivariate logistic regression model. No significant association was observed between all comorbidities in CCI and rs73064425, except cerebrovascular disease (*P* = 0.03, Table 3). We performed stratified analysis according to rs73064425 to assess the association between CCI and COVID-19 severity among different strata, finding that the associations were consistent with the wild (Genotype CC) and mutant (Genotype CT/TT) group (Table S2).

**Table 3:** Association between rs73064425 with age and comorbidities included in CCI

Outcome	OR^*^	95%CI	*P* Value^*^
Age	0.98	0.71–1.35	0.90
Myocardial infarction (MI, %)	0.98	0.79–1.22	0.86
Congestive heart-failure (CHF, %)	1.11	0.82–1.50	0.48
Peripheral vascular disease (PVD, %)	1.12	0.87–1.45	0.38
Cerebrovascular disease (CVD, %)	0.75	0.58–0.97	0.03
Dementia (%)	1.09	0.68–1.78	0.71
Chronic pulmonary disease (CPD, %)	1.06	0.93–1.20	0.36
Rheumatic diseases (Rh, %)	0.97	0.72–1.31	0.86
Peptic ulcer disease (PUD, %)	0.90	0.68–1.19	0.47
Mild liver disease (MLD, %)	0.90	0.66–1.24	0.53
Diabetes (DM, %)	0.91	0.77–1.08	0.29
Hemiplegia / Paraplegia	1.12	0.72–1.73	0.61
Moderate or severe renal disease (SRD, %)	1.16	0.88–1.52	0.30
Diabetes with end-organ damage (DMD, %)	0.81	0.46–1.41	0.45
Any malignant tumor (MT, %)	0.95	0.82–1.11	0.54
Moderate or severe liver disease (SLD, %)	1.29	0.62–2.67	0.49
Metastatic solid tumor (ST, %)	0.95	0.64–1.41	0.79
AIDS/HIV	2.15	0.63–7.31	0.22

^*^Odds ratio and *P* value were estimated in the logistic regression model, adjusted for age, gender, income, BMI, and genotype batch

**Fig. 4:**
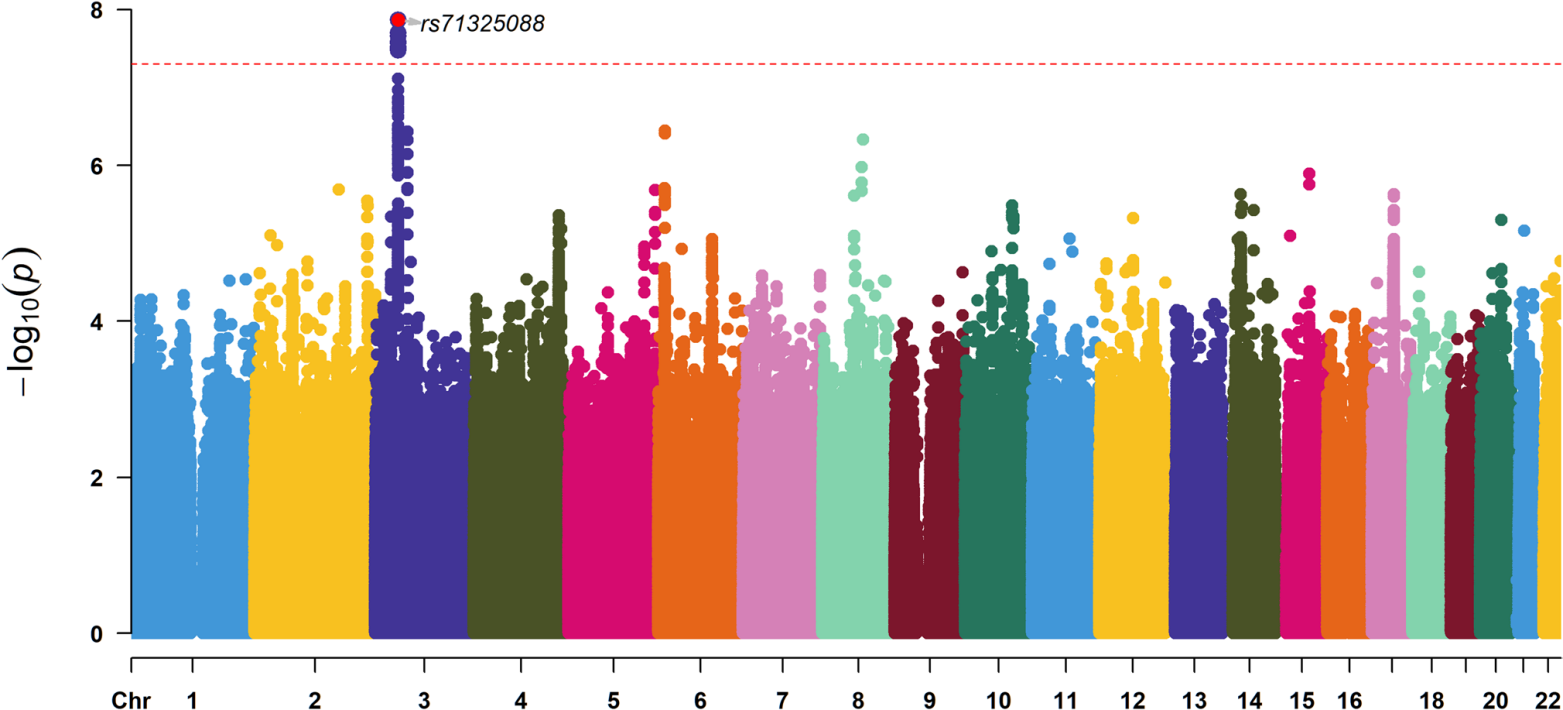
Result of Genome-wide association study in low CCI group matched by gender and age. (Lead SNP rs71325088 was in high LD with rs73064425, r.^2^ = 0.99)

Matched GWAS in the low CCI group found only one lead SNP rs71325088 at locus 3p21.31, in high LD with rs73064425 (*P* = 2.61 × 10^–8^, *r*^2^ = 0.99), reached a genome-association significant level of P < 5 × 10^–8^ (Fig. 4).

### Colocalization of cis-eQTL and COVID-19 GWAS Signals

**Fig. 5:**
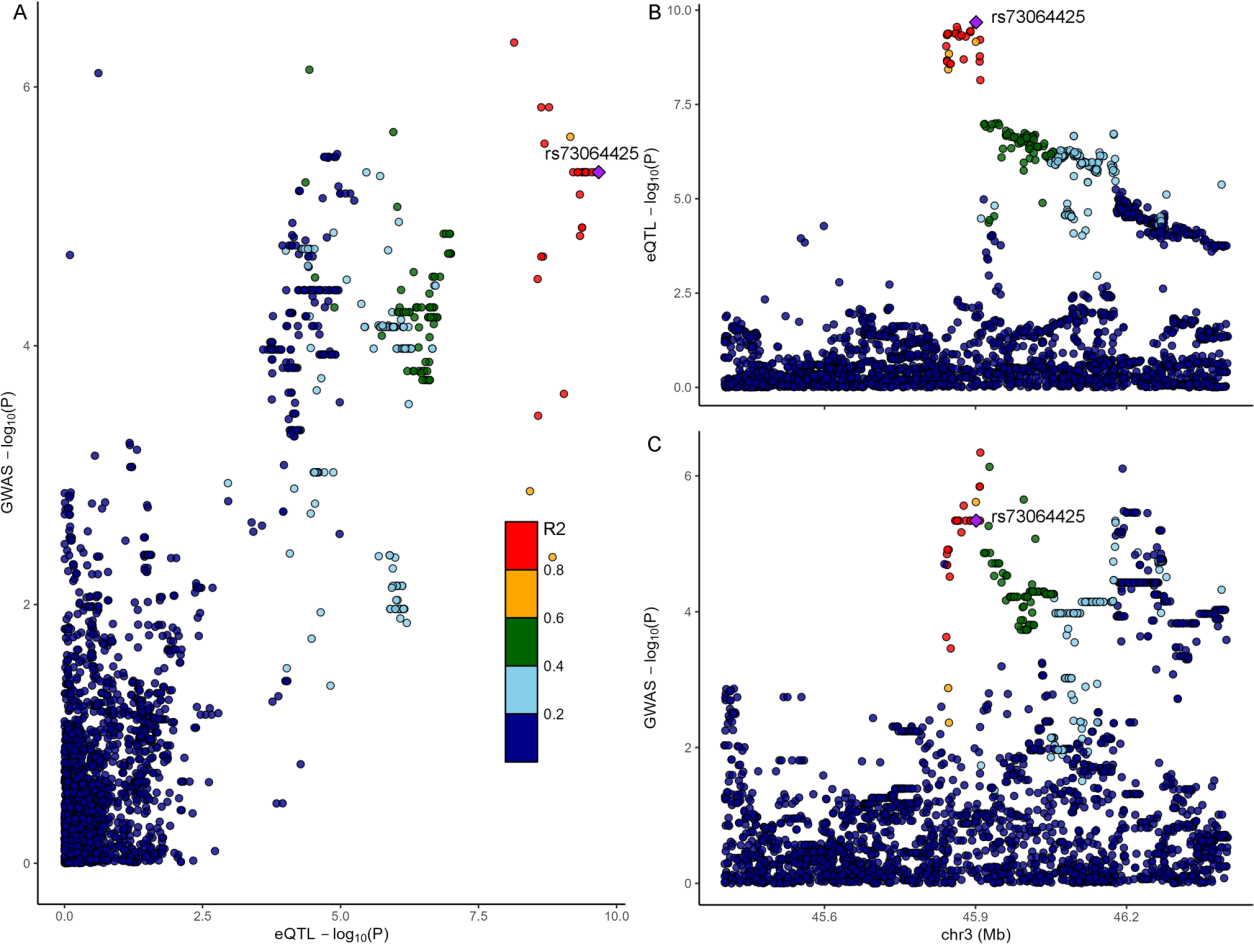
Colocalization of SLC6A20 gene expression and GWAS result in Esophagus Muscularis tissue. **A**: Scatterplot of GWAS P-Value and eQTL P-Value for shared variants; **B**, **C**: variants in LD with the lead SNP rs73064425

Then, colocalization analysis was performed between cis-eQTL signals of each gene in 49 tissues in the GTEx database and COVID-19 GWAS summary data to explore the association of gene expression and the variants using Coloc. rs73064425, associated with COVID-19 severity, was found to be colocalized with eQTLs for two genes, SLC6A20 and LZTFL1, with a posterior probability > 0.75. The eQTL signals of rs73064425 presented in four different tissues, including breast, esophagus muscularis, skeletal muscle, tibial nerve, for gene SLC6A20 (Fig. 5), and present in testis only for gene LZTFL1 (Table S3). The posterior probability of each SNP was calculated to determine the causal variant assuming one causal variant per trait in Coloc. The variant with the highest likelihood of causality was rs73064425, and all other SNPs with posterior probability over 0.75 were in high LD (*r*^2^ > 0.8) with rs73064425. Full results could be found in Supplement Fig S2.

### Polygenetic Risk Score and Predictive Model Construction

Considering the *R*^2^ and number of SNPs, we select *P* value threshold 5 × 10^–7^ to construct PRS including 86 independent SNPs (Table S4, Table S5). We divided the individuals into two groups by the low (bottom 50%) and high (top 50%) PRS risk group (Fig S3) and predicted the prognosis in a multivariable logistic regression model. The result shows that individuals with high genetic risk had a higher risk (OR = 1.57, 95%CI 1.32–1.87) of developing severe COVID-19 than the low genetic risk group (Table S6) adjusted for age, gender, income, BMI, CCI, and first 10 PCs.

**Fig. 6:**
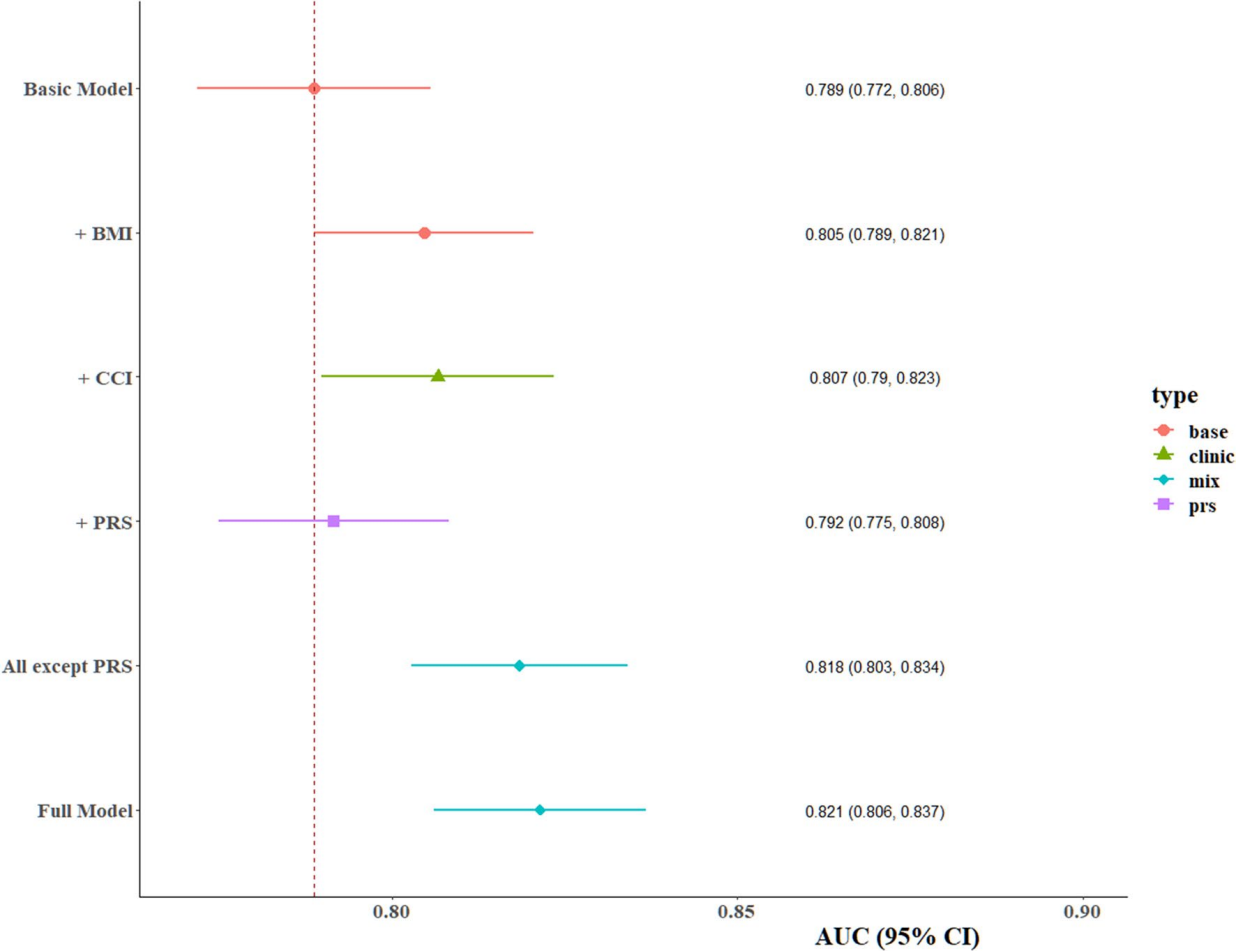
Prediction of risk of severe disease among cases with COVID-19 in the UK Biobank cohort based on demographic characteristics, comorbidities, and host genetic factors

Then, we constructed a predictive model for COVID-19 severity by involving demographic characteristics, comorbidities and PRS as predictors in the training set using four machine learning methods (Logistic regression, random forest, bagging FDA, and PLS). AUROC were close between different algorithms, ranging from 76.6 to 82.4% for the full model in the training set (Table S7). In the validation set, the logistic regression model was selected to report considering its simplicity and high performance. AUROC of the basic model including sex, age, and income reached 78.9% (Fig. 6, Table 4). The PRS could improve the AUROC by 0.3%, while the largest improvement to the model was the inclusion of CCI (AUROC = 80.7%, 95%CI 79.0–82.3%). The full model achieved the highest predictive power (AUROC = 82.1%, 95%CI 80.6–83.7%). We also calculated the population attributable fraction (PAF) [39], an estimate of the proportion of events that theoretically would not have occurred if all individuals would have been in the low-PRS and low-CCI group. Genetic was estimated to explain 18.2% (95% CI 13.3–23.2%) of the population’s risk of developing severe COVID-19, suggesting nearly 20% of events would have been prevented if all individuals were at low genetic risk. The contribution of comorbidities to the risk of severe COVID-19 was comparable to genetics, with the PAF estimated as 21.4% (95% CI 18.1–24.7%). And 39.7% (95% CI 34.0–45.5%) severe cases would not have occurred if all infected people were free of comorbidities and in low genetic risk.

**Table 4:** The performance of predictive models in validation set

Models	Variables	AUROC	Sensitivity	Specificity
Logistic regression	Basic model^*^	0.789	0.779	0.665
	+ BMI	0.805	0.779	0.679
	+ CCI	0.807	0.710	0.756
	+ PRS	0.792	0.776	0.679
	All except PRS	0.818	0.737	0.747
	Full Model	0.821	0.768	0.722

*Basic model were constructed with age, gender, and income as predictors

## Discussion

In this study, we assessed the effect of different factors on the risk of severe COVID-19 by comparing the difference in demographic characteristics and comorbidities between the severe case and non-severe case groups. Similar to previous studies [4, 11, 40–42], we found that elder, male, low economic status, high BMI, and comorbidities were risk factors for COVID-19 severity. In the GWAS analysis, we find one independent genetic association (rs73064425) with severe COVID-19. Furthermore, rs73064425 located at 3p21.31 was found to affect the cis-regulation expression of two genes (LZTFL1 and SLC6A20) in the colocalization analysis. Additionally, we performed a GWAS in low-CCI participants matched by gender and age and found consistent results as above.

The main difference between the present study and previous COVID-19-related GWAS studies is the selection of the control, where we chose the non-severe COVID-19 cases as the control, while most previous studies used the SARS-CoV-2 negative or unknown population as the controls [14, 16, 18, 19]. Thus, the loci found in this study actually reflected the risk of severe illness after infection, while by contrasting to generally SARS-CoV-2 negatives, the results were a mixture of susceptible loci for both infection and prognosis. This also partly explains that the previously published locus using UKB data was not fully replicated in our study.

Age played an important role in developing severe COVID-19 in our analysis, with the highest odds ratio in over 80 years group compared to the 50 ~ 60 group. A large-scale study found that the median age of patients who died from COVID-19 was 79 years old [43]. Male patients had 1.15-fold more risk progressed to severe disease than female, which may mainly due to gender-special behaviors, genetic and hormonal factors as Tu et al. [44] reported. Similar to the results 7 of other studies, comorbidities such as diabetes, liver disease and malignancy predispose to poor prognosis in patients with COVID-19 [7–11]. To assess the interaction between a genetic variant with age and comorbidities on COVID-19 severity, we performed association and stratification analysis, finding no significant interaction between them.

Downes et al. [45] found that leucine zipper transcription factors 1 (LZTFL1) can regulate epithelial-mesenchymal transition (EMT) related signaling and they identified lung epithelial cells undergoing EMT in lung tissue as a possible cause of susceptibility to severe COVID-19 associated with 3p21.31. Another potentially causal gene is SLC6A20 that may be responsible for the increased risk of poor prognosis. It encodes the sodium–imino acid (proline) transporter 1 (SIT1), which functions as a proline transporter expressed in the kidney and small intestine [46]. Raphael et al. [47] found that SIT1functionally interacts with angiotensin-converting enzyme 2 (ACE2), which has been demonstrated in many studies to be a receptor on the surface of SARS-CoV-2 invading cells [14, 48–51]. SLC6A20 was found to be affected by rs73064425 in esophagus tissue, where the ACE2 was expressed [52]. Other tissues such as skeletal muscle and tibial nerve were associated with symptoms subsequent to SARS-Cov-2 infection [53, 54], which may be the mechanism responsible for severe COVID-19.

Meanwhile, we can assume that rs73064425 and its related gene (LZTFL1, SLC6A20) are playing an important role in both susceptibility and severe disease since this is a common locus in both types of studies that utilizing different sources of controls.

In the present study, we constructed a polygenic risk score representing an individual genetic risk for severe COVID-19 based on GWAS summary data from a prior study. In line with previous studies [14], our study suggested that higher genetic risk increases the risk of adverse outcomes. The PRS would help stratifying the population into subgroups with different risk levels to alert clinical and individual decision-making in advance.

Furthermore, we developed predictive models based on multiple risk factors of both genetic and non-genetic identified in our study to assess the risk of developing critical illness after being infected with SARS-CoV-2 and validated it in the internal validation set. Four different algorithms were applied to develop the predictive models. The final model (logistic regression) is simple with easily assessable variables and highly interpretable. The results showed that the predictive model including demographic characteristics (age, gender, BMI, and income), comorbidities (CCI) and genetic risk (PRS) could well identify people at high risk of severe COVID-19 with an AUROC of 82.1%, which was comparable with previous models [14, 55]. Genetic and comorbidities contributed 18.2% and 21.4% to severe COVID-19 risk, emphasizing that COVID-19 patients with high genetic risk and underlying disease should be taken more care of in clinical practice to deal with disease progression in advance.

### Strength and Limitation

The main strengths of our present study compared with prior studies are that we focus on the influence of host genetic factors on COVID-19 severity rather than incidence because as a communicable disease, pathogenic infections are one of the key factors in disease progression. And we further analyse the potential interaction of genetic variant and comorbidities on COVID-19 severity. In addition, we develop a predictive model with AUROC as high as 80% using only six easily assessable variables, which could be used in clinical practice. Our study also has a few limitations though. First, all COVID-19 patients in our study were over 50, which may limit the generalization of the study conclusions in young patients. Second, although the known potential confounders were adjusted in our analysis, it is possible that unmeasured confounders and bias remained, such as vaccination and virus strains. Third, most participants in the UK Biobank cohort were unknown of the SARS-CoV-2 test, which may influence the prevalence of severe COVID-19 and underestimate the effect of risk factors.

## Conclusion

In this study, rs73064425 and its two colocalized genes, LZTFL1 and SLC6A20 were found to be associated with COVID-19 severity. The logistic regression model was developed to predict prognosis of COVID-19 patients early and could be used in clinical practice. Our findings demonstrate the need for clinical care of patients with comorbidities and high genetic risks. From the public health perspective, prevention should be enhanced in the elderly and in people with underlying diseases and high genetic risks, who often suffer critical conditions after infection. More researches are needed to explore the mechanism of comorbidities on the risk of severe COVID-19 in the future, especially in people with high genetic risk.

## Supplementary Information

Below is the link to the electronic supplementary material.

Supplementary file1 (DOCX 595 KB)

Supplementary file2 (XLSX 32 KB)

## Data Availability

Data from the UK Biobank (http://www.ukbiobank.ac.uk/) are available to all researchers upon making an application. Part of this research was conducted using the UK Biobank Resource under Application 92718.
