# Supplementary material for: Host Genetic Factors, Comorbidities and the Risk of Severe COVID-19
Source: J Epidemiol Glob Health. 2023 May 9;13(2):279–91. doi: 10.1007/s44197-023-00106-3 (PMC10169198; doi:10.1007/s44197-023-00106-3)

**Supplementary**

1. **Fig S1: Distribution of CCI in all COVID-19 patients**

**
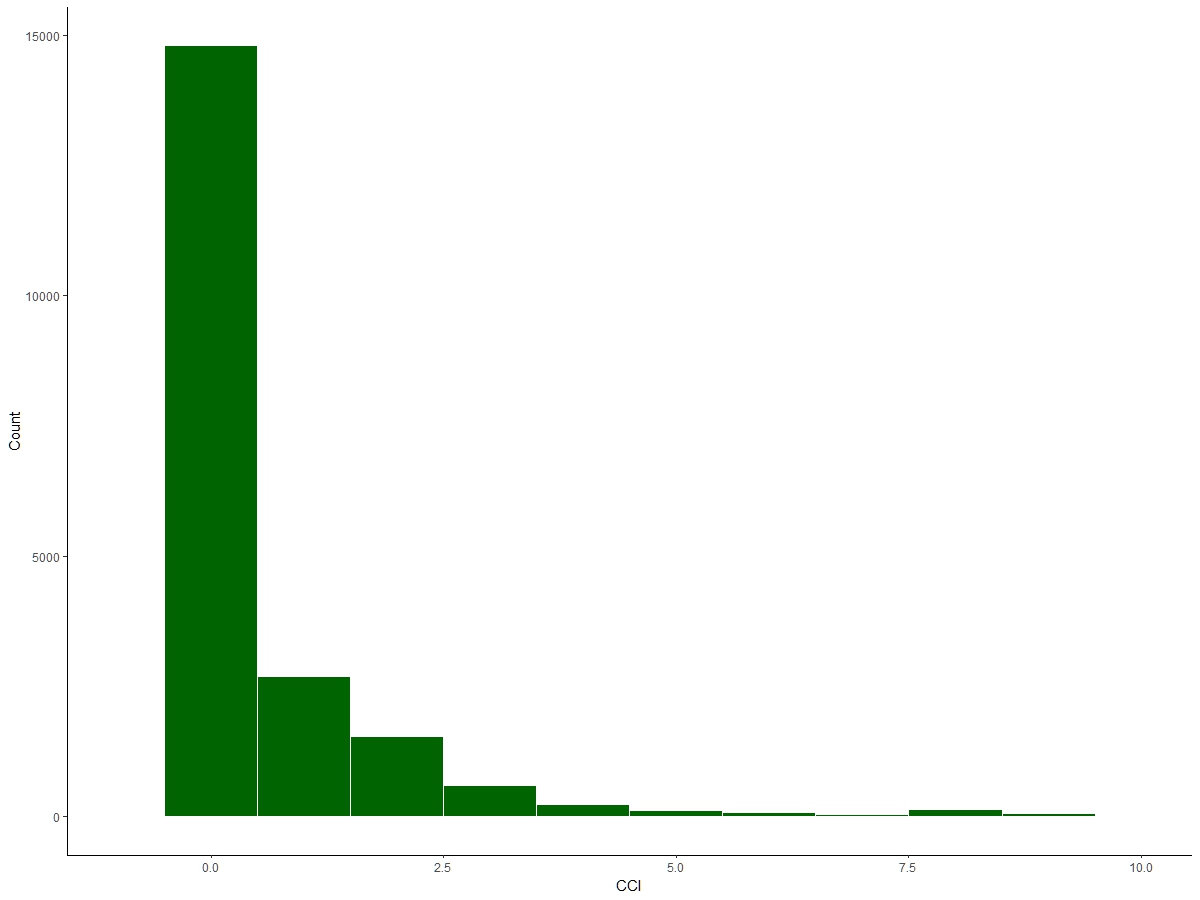
**

1. **Fig S2: Colocalization of eQTL and GWAS pairs**

A: SLC6A20 region in Breast Mammary Tissue; B: SLC6A20 region in Esophagus Muscularis tissue; C: SLC6A20 region in Muscle Skeleta tissue; D: SLC6A20 region in Nerve Tibial tissue; E: LZTFL1 region in Testis tissue


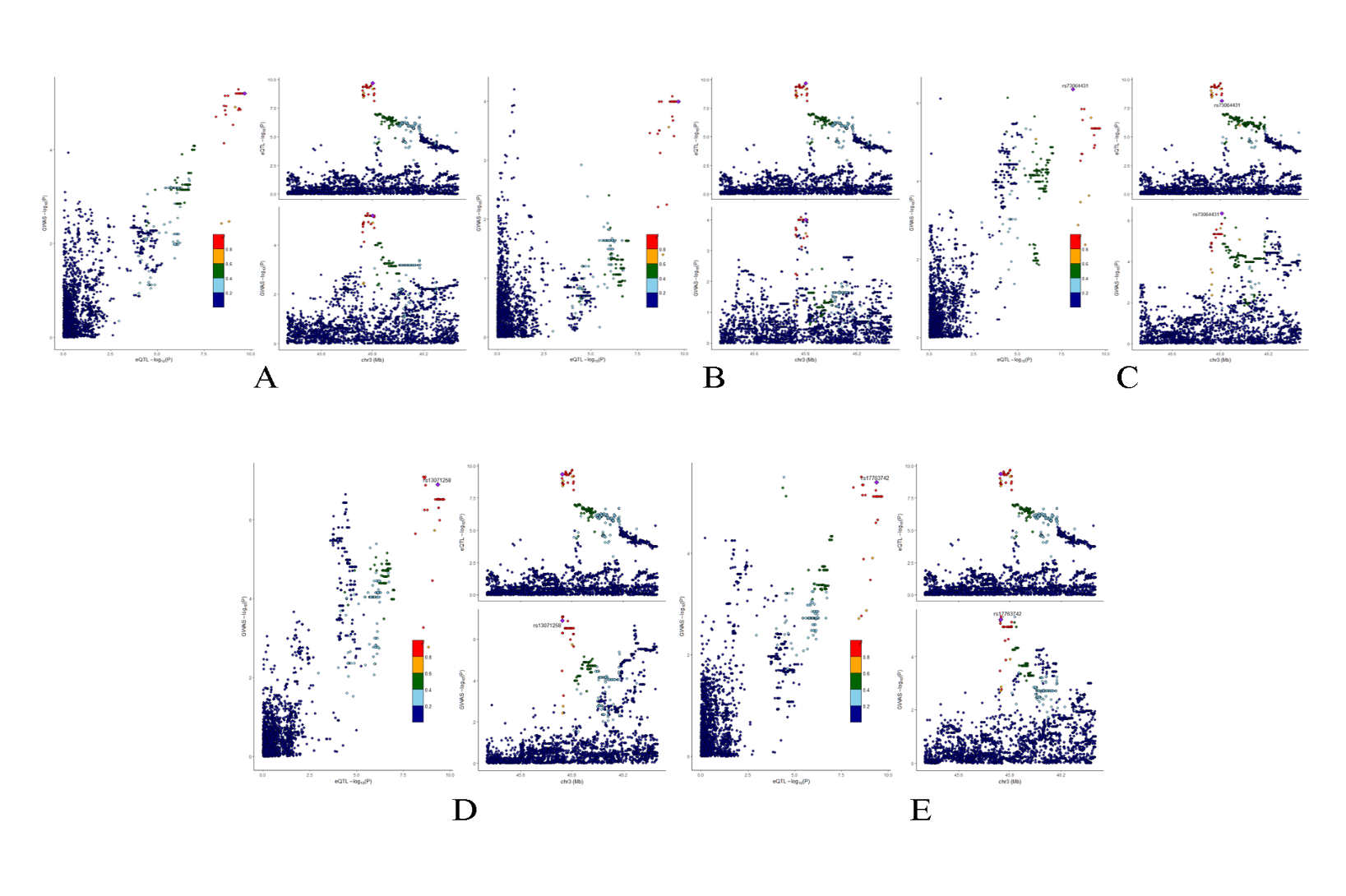


1. **Fig S3: Distribution and classification of PRS in COVID-19 cases**


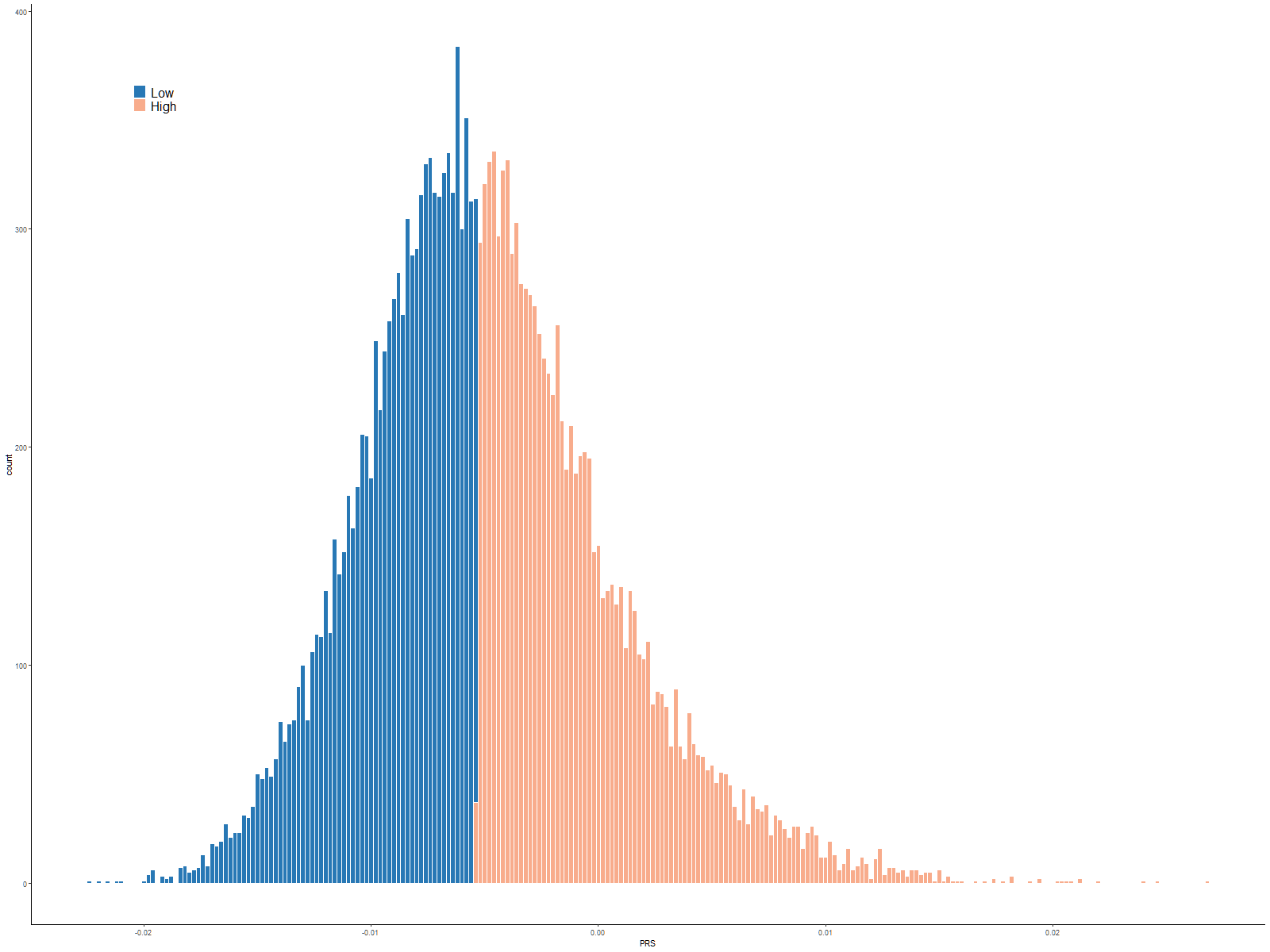

Supplement: Supplementary file 1 — Supplementary file1 (DOCX 595 KB) [file 44197_2023_106_MOESM1_ESM.docx]
